# SPIONs Prepared in Air through Improved Synthesis Methodology: the Influence of γ-Fe_2_O_3_/Fe_3_O_4_ Ratio and Coating Composition on Magnetic Properties

**DOI:** 10.3390/nano9070943

**Published:** 2019-06-28

**Authors:** Joana C. Matos, M. Clara Gonçalves, Laura C. J. Pereira, Bruno J. C. Vieira, João Carlos Waerenborgh

**Affiliations:** 1 Centro de Química Estrutural, Universidade de Lisboa, Av. Rovisco Pais, IST, 1000 Lisboa, Portugal; 2 Centro de Ciências e Tecnologias Nucleares, Instituto Superior Técnico, Universidade de Lisboa, 2695-066 Bobadela LRS, Portugal; 3 Departamento de Engenharia Química, Instituto Superior Técnico, Universidade de Lisboa, Av. Rovisco Pais, IST, 1000 Lisboa, Portugal

**Keywords:** SPIONs, magnetite, maghemite, coating, PEG, dextran

## Abstract

Superparamagnetic iron oxide nanoparticles (SPIONs) have shown great potential in biomedicine due to their high intrinsic magnetization behaviour. These are small particles of magnetite or maghemite, and when coated, their surface oxidation is prevented, their aggregation tendency is reduced, their dispersity is improved, and the stability and blood circulation time are increased, which are mandatory requirements in biomedical applications. In this work, SPIONs were synthesized in air through a reduction-precipitation method and coated with four different polymers (Polyethylene glycol(PEG) 1000/6000 and dextran T10/T70). All the synthesized samples were structurally and magnetically characterized by transmission electron microscopy, Fourier transform infra-red spectroscopy, X-ray powder diffraction, Mössbauer spectroscopy, and Superconducting Quantum Interference Device (SQUID) magnetometry. SPIONs centrifuged and dried in vacuum with an average diameter of at least 7.5 nm and a composition ≤60% of maghemite and ≥40% of magnetite showed the best magnetization results, namely a saturation magnetization of ~64 emu/g at 300 K, similar to the best reported values for SPIONs prepared in controlled atmosphere. As far as SPIONs’ coatings are concerned, during their preparation procedure, surface polymers must be introduced after the SPIONs’ precipitation. Furthermore, polymers with shorter chains do not affect the SPIONs’ magnetization performance, although longer chain polymers significantly decrease the coated particle magnetization values, which is undesirable.

## 1. Introduction

Superparamagnetic iron oxide nanoparticles (SPIONs), the only clinically approved metal oxide nanoparticles (NPs) [1], emerge as medically interesting nanoparticles (NPs), being able to integrate multifunctional platforms and to perform multiple objectives such as imaging and therapy, (theranostics) or to perform a single advanced function through the incorporation of multiple functional units [2,3,4,5,6,7,8,9]. Concerning biomedical applications, SPIONs may be externally magnetically triggered, endowing a wide variety of purposes, namely: (i) site-specific magnetic targeting [10], (ii) target drug delivery of drugs and genes [11,12], (iii) negative contrast agents in magnetic resonance imaging (MRI) [13,14], (iv) hyperthermia treatment under alternating magnetic fields [15,16], (v) magnetic transfections [17], (vi) iron detection [18], (vii) chelation therapy [19], (viii) tissue engineering [20], (ix) stem cell tracking [21] and (x) cell separation and isolation [22].

When intravenously injected (the fastest route of drug administration in humans/mammals), the biological performance of NPs is controlled by a complex array of interrelated physicochemical and biological factors, starting with *opsonization* [23,24,25,26,27]. Here the NP becomes covered with biologic proteins (opsonins), forming a coating thereby conferring a new biological identity, which determines the subsequent cellular/tissue response. The composition of protein corona is strongly dependent on the physicochemical properties of NPs. Hydrophobic surface NPs are, in general, rapidly and efficiently coated with plasma components (corona/opsonins) being rapidly removed from the mammal’s circulation system (by phagocytose and clearance), while some hydrophilic NPs are able to resist this immune system elimination process, for example, those which are coated with polymers such as polyethylene glycol (PEG) or dextran [27,28,29]. Therefore, it is mandatory to modify the common SPIONs’ hydrophobic surface character in order to overcome this limitation. To produce hydrophilic and biocompatible SPIONs, two different routes are usually pursued: ligand exchange and encapsulation with amphiphilic polymers. The ligand exchange consists of the substitution of native hydrophobic surfactants by hydrophilic ligands. The encapsulation process involves an amphiphilic block copolymer or inorganic shell to accommodate one or more SPIONs within the liposomal structural or core/shell NPs, respectively [25]. SPIONs coating is important since (i) it decreases the SPIONs high aggregation tendency, (ii) it protects SPIONs from surface oxidation, and thus (iii) increases their blood circulation time. SPIONs stabilization with a hydrophilic polymer chain opens the door to new opportunities. The most commonly used SPIONs coatings are derivatives of dextran, polyethylene glycol (PEG), polyethylene oxide (PEO) poloxamers and polyoxamines [30,31]. However, it is important to mention that this surface coating, depending on its nature, amount, composition and thickness, may affect the inherent magnetic properties of SPIONs.

SPIONs’ size is another final parameter in the capability to reach and interact with target cells [32,33]. Administration route and SPIONs’ surface properties dictate their ultimate effect in terms of the efficiency of cellular uptake, biodistribution, metabolism, and potential toxicity [27,28,34].

In the present work, a new SPIONs synthesis methodology was developed, improving the classical synthesis path (co-precipitation method). All the SPIONs were prepared in air at room temperature, followed by different separation and drying approaches. The synthesis parameters were optimized so that the inherent properties of the obtained SPIONs (naked and coated) were perfectly reproducible between syntheses. In this paper, the results concerning different samples of SPIONs (naked SPIONs and coated SPIONs) will be presented and discussed. SPIONs were coated with four different polymers (PEG 1000/6000 and dextran T10/T70).

In order to determine the composition, NPs size and magnetic behaviour, all the synthesized SPIONs samples were characterized by transmission electron microscopy (TEM), Fourier transform infrared spectroscopy (FTIR), powder X-ray diffraction (XRD), Mössbauer spectroscopy and SQUID magnetometry. Two different magnetic behaviours have been identified as dependent on SPIONs’ dimensions.

## 2. Materials and Methods 

### 2.1. Materials

Ferric chloride hexahydrate (FeCl_3_·6H_2_O) and hydrochloric acid (HCl) were purchased from Sigma-Aldrich (Darmstadt, Germany). The anhydrous sodium sulphite (Na_2_SO_3_) and the ammonium hydroxide (NH_4_OH, 25% *w*/*w*) were purchased from Merck (Darmstadt, Germany) and Scharlau (Barcelona, Spain), respectively. Polyethylene glycol (PEG) 1000/6000 were purchased from Merck and dextran T10/T70 from Pharmacia (Stockholm, Sweden). The deionized water was obtained in Centro de Ciências e Tecnologias Nucleares facilities.

### 2.2. Experimental Methodology 

#### 2.2.1. SPIONs Synthesis

SPIONs (Samples A) were synthesized through optimization of the reduction-precipitation method based on previous team work [35]. All experimental parameters were revised and the final drying step was enhanced through a freeze-drying process.

Briefly, 16.7 mmol of FeCl_3_·6H_2_O were dissolved in 2.5 mL of acidic medium of HCl 2 M and 2.5 mL of deionized water (solution 1). At the same time, 3.3 mmol of Na_2_SO_3_ were dissolved in 3 mL of deionized water (solution 2). Then, the two solutions (1 and 2) were mixed (forming new solution 3), and an immediate change in colour was observed, from yellow to dark red. In this method, Na_2_SO_3_ is the reducing agent, reducing Fe^3+^ to Fe^2+^ in air at room temperature. Solution 3 was then added to NH_4_OH (452.2 mM) solution, under intensive magnetic stirring, promoting the precipitation of a dark compound. Different washing and drying procedures were attempted in order to optimize the synthesis protocol. Thus, the precipitate was centrifuged twice with deionized water 3000 rpm during 15 min (sample A1.1) or just separated from the reactional bath by sedimentation and then washed four times with deionized water (sample A2.1). In both cases (A1.1 and A2.1), the precipitate was dried in air. Alternatively, the centrifuged precipitate was dried in vacuum (sample A3.2). Table 1 summarizes the SPIONs samples acronyms.

#### 2.2.2. SPIONs Coating

SPIONs were coated with different polymers (PEG 1000/6000 and dextran T10/T70), following two different methods.

In method 1 (samples B), polymers were introduced during the synthesis process, immediately before the precipitate formation. The polymers were dissolved in the ammonium solution under vigorous magnetic stirring, and after its complete dissolution, solution 3 was added leading to the precipitate formation.

In method 2 (samples C), polymers were introduced after the precipitate formation. In this case, SPIONs were synthesized as described in the previous Section 2.2.1 and their coating was made as follows: 800 mg of SPIONs were suspended in 10 mL of 0.5 M NaOH and 4% *w*/*w* of polymer (PEG 1000/6000 or dextran T10/T70) was dissolved in 10 mL of 0.5 M NaOH. After this, the SPIONs suspension was added slowly to the polymer solution under intensive magnetic stirring and left for 24 h in magnetic stirring. Finally, the coated SPIONs were washed four times with deionized water and freeze dried during 48 h. Table 2 summarizes the coated SPIONs samples acronyms.

#### 2.2.3. Characterization Techniques

##### **Transmission Electron** **Microscopy (TEM)**

Morphology and size (static diameter) of naked and coated SPIONs were determined by transmission electron microscopy, TEM. The size measurements were made with a minimum of 100 independent measurements *per* sample, based on a simple random sample without the replacement method. The static diameter was obtained using the measure tool from Image J (National Institute of Mental Health, Bethesda, MD, USA), after proper calibration. 

To prepare a TEM sample, a drop of suspension was placed on a copper grid and dried at room temperature. A Hitachi H-8100 model (Chiyoda, Tokyo, Japan) was used and the micrographs were obtained using an applied tension of 200 KV. This model is a conventional TEM with a high brightness LaB6 electron source and large specimen tilt (>30°) capabilities.

##### **Fourier Transform** **Infrared (FTIR) Spectroscopy**

FTIR analysis of naked and coated SPIONs was performed with potassium bromide pellets (KBr, ≥99%, FTIR grade, from Sigma-Aldrich (Darmstadt, Germany)). The SPIONs samples were finely ground and mixed with potassium bromide and then pressed into a disc (5 mg of SPIONs and 200 mg of KBr). A KBr pellet was used as background. 

Nicolet 5700 model from Thermo Electron Corporation (Waltham, MA, USA) was used in transmission mode, through a KBr beam splitter.

##### **Powder X-ray** **Diffraction (XRD)**

The crystal structure and phase purity of naked and coated SPIONs were analyzed by powder X-ray diffraction (XRD). The diffractograms were obtained with a PANalytical X’Pert Pro diffractometer, (The Bruel and Kjaer Building, Jarman Way, Royston, SG8 5BQ United Kingdom), using Cu Kα radiation. Data were collected in steps of 0.02° in the 20°–80° range (2 theta) with a counting time per step of 4 s. The cell parameters were calculated by the Rietveld method using the FullProf software [36].

##### **Mössbauer** **Spectroscopy**

The Mössbauer spectra of naked and coated SPIONs were collected in transmission mode using a, conventional constant-acceleration spectrometer (Wissel, Würmstrasse 8, D-82319, Starnberg, Germany) and a 25 mCi ^57^Co source in the Rh matrix. The velocity scale was calibrated using α-Fe foil. The estimated isomer shifts, IS, are given relative to this standard at room temperature. The absorbers were obtained by packing the powdered samples into Perspex holders (~5 mg/cm^2^ of natural Fe). Low-temperature spectra were collected using a bath cryostat with the sample immersed in liquid He. The spectra were fitted to Lorentzian lines using a non-linear least-squares method [37], while necessary distributions of magnetic splittings were fitted according to the histogram method [38].

##### **Magnetization** **Measurements**

DC magnetic properties of the naked and coated SPIONs samples were measured using a 6.5T SQUID magnetometer (S700X; Cryogenic Ltd., Acton Park Industrial Estate, The Vale, London, UK). The zero-field cooled (ZFC) and field-cooled (FC) measurements were performed by cooling the sample to 5 K at zero field or in the presence of an external field of 25 Oe, respectively. All the magnetic measurements were carried out at increasing temperatures within the range 5–320 K. Isothermal magnetization curves were obtained for magnetic fields up to 5 T at 10 and 300 K.

When appropriate, AC susceptibility measurements were also performed using the multi characterization MagLab2000 System (Oxford Instruments, Abingdon, Oxfordshire, UK). The in-phase (χ′) and out-of-phase (χ″) linear susceptibilities were measured at two different frequencies, 995 and 4995 Hz, in the temperature range 5–300 K with an AC driving field of 5 Oe.

## 3. Results

### 3.1. Size, Morphology and Structural Analysis

**Figure 1:**
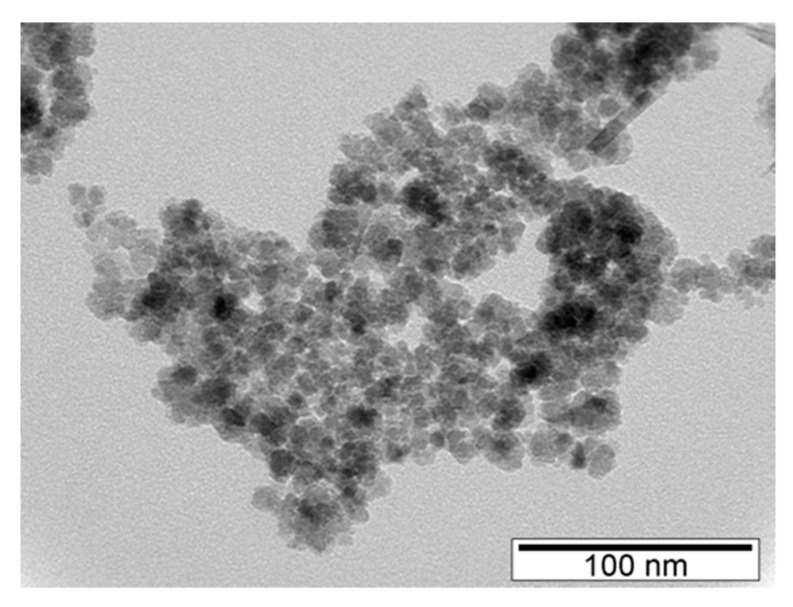
TEM micrograph of naked SPIONs: A 1.1.

TEM micrographs concerning naked SPIONs (samples A, Figure 1) show nearly spherical-shaped particles with static diameters between 10.5 nm for sample A 1.1 and 7.5 nm for sample A 3.2 (Table 3). Although the difference between the diameters is small, this observation allows us to conclude that the synthesis process, particularly the washing step, has a role in determining the size of the synthesized NPs. 

TEM micrographs of the coated SPIONs prepared by method 1 (samples B) show a large decrease in particles sizes (Figure S1, see Supplementary Material). This fact is attributed to an early introduction of polymers during the SPIONs synthesis: Just after the nuclei formation, the polymers are adsorbed on the nuclei surfaces hindering the particles’ growth. The obtained static diameters are approximately 4.4 nm and 1.1 nm, for samples B1.1 and B2.2, respectively.

**Figure 2:**
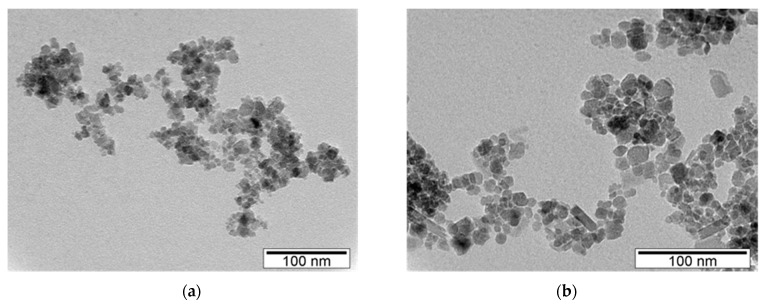
TEM micrographs of coated SPIONs: (**a**) C 1.1, (**b**) C 2.1.

Coated SPIONs synthesized by method 2 (samples C, Figure 2) present a diameter of ~8 nm similar to the diameter observed for sample A 3.2 synthesized according to the same synthesis protocol. Furthermore, since the polymer coatings are not observable by TEM, the sizes of C and A3.2 samples were expected to be identical, confirming experimental synthesis reproducibility.

**Figure 3:**
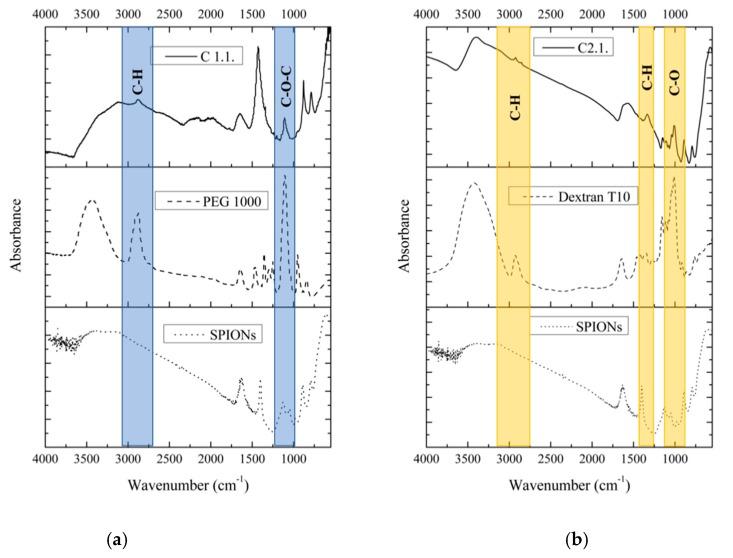
FTIR spectra of coated SPIONs: (**a**) sample C 1.1 and (**b**) C 2.1.

To prove that SPIONs were effectively coated, FTIR characterization of the C samples was carried out (Figure 3). The naked SPIONs spectrum shows a broad band at ~3500 cm^−1^, attributed to the presence of surface hydroxyl groups and two bands centered at ~453 cm^−1^ and 586 cm^−1^, characteristic of the Fe-O bond [39]. The polymers spectra exhibited characteristic bands of PEG (~3400 cm^−1^, 2900 cm^−1^, ~1450–1292 cm^−1^, ~1250 cm^−1^, 1100–1060 cm^−1^) [40] and dextran (~3400 cm^−1^, ~2900 cm^−1^, ~1600 cm^−1^, ~1250–1460 cm^−1^, ~1040–1150 cm^−1^) [41].

In SPIONs coated with PEG, samples C 1.1 (Figure 3a) and C 3.1, the naked SPIONs peaks are predominant while the characteristic peaks of PEG are strongly decreased. However, the FTIR spectra clearly show the main peaks of PEG centered at ~2900 cm^−1^, attributed to the C–H stretching, and at ~1110 cm^−1^, attributed to the stretching of C–O–C groups.

In the case of dextran coating samples C 2.1 (Figure 3b) and C 4.1, the characteristic vibrations of C–H at ~ 2900 cm^−1^, C–H bending at ~ 1330 cm^−1^ and the stretching vibration of C–O groups at ~1020 cm^−1^ are observed in the FTIR spectra in addition to the naked SPIONs peaks.

No new peaks that might correspond to bonds established between SPIONs and PEG or dextran are observed. However, the presence of the strongest peaks of these polymers in the FTIR spectra of the C samples indicates that the surface of these SPIONs are covered by them.

### 3.2. XRD and Mössbauer Characterization

**Figure 4:**
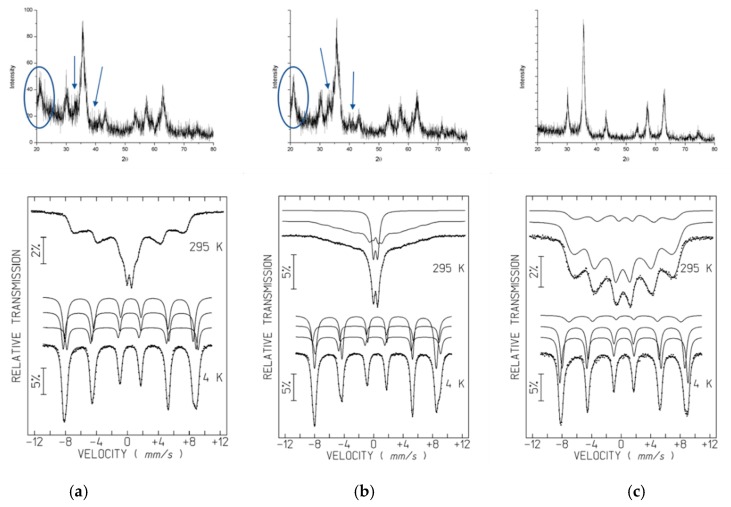
XRD diffractograms and Mössbauer spectra of naked SPIONs: (**a**) A 1.1, (**b**) A 2.1, (**c**) A 3.2.

XRD diffractograms of the A samples (A1.1, A2.1, A3.2) (Figure 4) show broad diffraction peaks consistent with nano-sized crystallites, allowing the identification of the main peaks of a spinel phase. Unit-cell parameters estimated from the FullProf program are between those of magnetite with ideal stoichiometry Fe_3_O_4_ (JCPDS file 19–629) and maghemite γ-Fe_2_O_3_ (JCPDS file 39–1346). Fe_3_O_4_ and γ-Fe_2_O_3_ are isostructural. As confirmed below by Mössbauer spectroscopy in the A samples, magnetite is partially oxidized. There is no reason to believe that all the spinel grains have exactly the same oxidation degree. Most probably magnetite grains with different oxidation degrees up to the maghemite limit where all the Fe cations are in the 3+ oxidation state, are present in the studied samples leading to a mixture of spinel grains with different unit-cell parameters. This further broadens the XRD peaks, in addition to the small grain size effect. An additional peak at 0.418 nm, highlighted by ellipsoids on Figure 4, is clearly observed for A1.1 and A2.1. Together with peaks at 0.269 nm and 0.219 nm (absent for A3.2), the 0.418 nm peak suggests the presence of goethite. The remaining diffraction peaks of this oxyhydroxide phase overlap the most intense spinel peaks. The presence of goethite is confirmed by Mössbauer data of A1.1 and A2.1 at 4 K (see below).

The Mössbauer spectra of the A samples at room temperature, showing sextets with very broad peaks, and in the case of A2.1, an additional doublet, is consistent with superparamagnetic iron oxides. These spectral features arise when crystallites are nanosized. They are so small (≤12 nm, depending on the Fe oxyhydroxide) that thermally-induced energy fluctuations change the direction of the magnetization of the NPs from one easy axis to another with a frequency higher than the Larmor precession frequency of the nuclear magnetic moment in the local field [42]. If NPs with a finite range of volumes are present, they will give rise to a superposition of spectra with different relaxation times.

Decreasing the temperature to 4 K slows down the relaxation of the direction of the magnetic moments and allows the observation of sharp peaks (Figure 4) even for the smallest iron oxyhydroxide NPs. The 4 K spectra of the A samples, nevertheless, cannot be fitted by a single sextet. Three sextets are necessary to adequately analyze the spectra. In the case of A1.1 and A2.1, the estimated parameters (Table 4) suggest that the sextets are due to Fe^3+^ in goethite and to tetrahedrally and octahedrally coordinated Fe^3+^ in maghemite [43]. Sample A3.2, on the other hand, instead of goethite, shows a sextet with higher isomer shifts consistent with Fe^2+^ in magnetite [44].

The room temperature spectra of the A samples were fitted with distributions of magnetic hyperfine fields, B_hf_, in order to simulate the magnetic relaxation signal [45]. One additional doublet was considered in the case of A2.1. The estimated isomer shifts, IS, are consistent with high-spin Fe^3+^. In the case of A3.2, where magnetite was identified in the 4 K spectrum, a refinement with two distributions of B_hf_ one assigned to Fe^3+^ and the other to Fe^2+^, similar to the analysis described by Gonçalves et al. [35] and Roca et al. [44], was performed. This analysis of the 295 K spectrum of A3.2 spectra leads to results fully consistent with those obtained for the 4 K spectrum. These results suggest that approximately 24% of the Fe was incorporated in magnetite, 16 % as Fe^2.5+^ and 8% as Fe^3+^ on the octahedral and tetrahedral sites, respectively, and the remaining Fe in maghemite.

The diffraction peaks of the coated B1.1 and B2.2 samples (Figure S2) are much broader than those in the diffractograms of the A samples. This agrees with the significantly smaller TEM NP size, < 4.4 nm, when compared to A particles, 7.5–10.5 nm (Table 3). The main X-ray bands are located at approximately 0.254 nm, 0.224 nm, 0.198 nm, 0.173 nm, 0.152 nm and 0.147 nm and may be attributed to the poorly ordered hydrous iron oxide ferrihydrite phase [46]. In B1.1, the 0.418 nm peak is also apparent, suggesting the presence of goethite. These results are also in agreement with Mössbauer data. At room temperature, the spectra consist of a single doublet while at 4 K the estimated parameters of two sextets agree with those reported for ferrihydrite (Figure S2, Table 4). For B1.1, a third sextet consistent with goethite is also observed.

**Figure 5:**
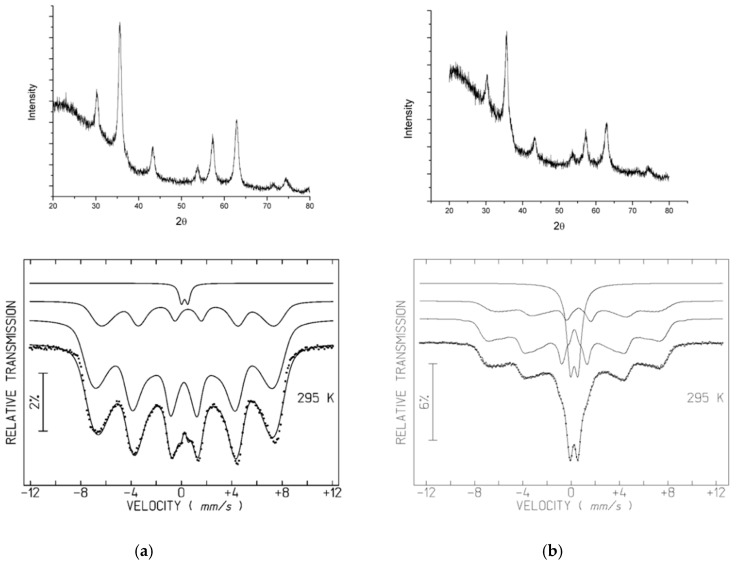
XRD diffractograms and Mössbauer spectra of coated SPIONs (Samples C): (**a**) C1.1, (**b**) C4.1.

In contrast with the coated B samples, the coated C samples were prepared by introducing the polymers after the precipitate formation, followed by washing and drying in vacuum. Changing the preparation step, where polymers are introduced, a dramatic effect occurs in the resulting material. In fact, the X-ray diffractograms and Mössbauer spectra of samples C (Figure 5) show the same phases as the naked A3.2 SPIONs, magnetite and maghemite. Neither goethite nor ferrihydrite are detected.

In spite of the similar average particle sizes, 7.5–8.5 nm (Table 3), the relative area of the doublet in the 295 K spectrum of sample C4.1 is higher than in the case of A3.2 and the remaining C samples coated with PEG 1000, PEG 6000 and Dextran T10 (Figure 4c and Figure 5b). A possible explanation is the steric hindrance caused by the significantly higher chain length of the T70 polymer when compared to the other polymers, which may increase the average distance between SPION crystallites. This fact weakens magnetic dipole interactions between SPIONs, responsible for the increase in fluctuations in the magnetization direction frequency of the C4.1, when compared to A3.2 or the remaining C samples.

### 3.3. Magnetic Properties Evaluation

**Figure 6:**
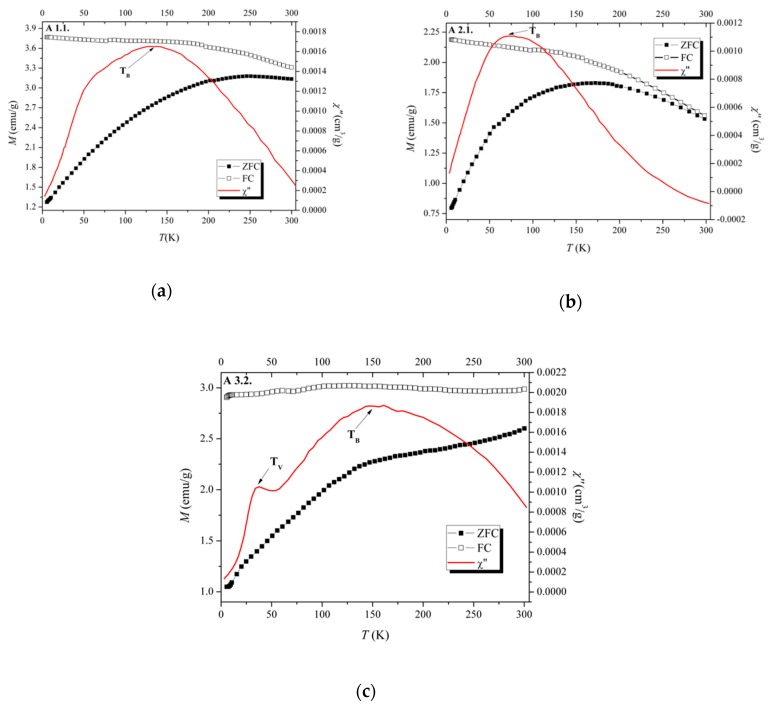
Temperature dependence of magnetization (ZFC/FC curves at 25 G) and AC susceptibility (out-of-phase component χ″ at 995 Hz) of naked SPIONs: (**a**) A 1.1, (**b**) A 2.1, (**c**) A 3.2.

The temperature dependence of magnetization for the naked SPIONs, samples A, is shown in Figure 6. An irreversibility of both ZFC and FC curves is observed with the ZFC curves showing broad maxima which indicate the appearance of a superparamagnetic state, typical for particles with such small dimensions. In sample A3.2, an additional kink at temperature lower than 100 K is probably related to the Verwey transition characteristic of magnetite. This temperature is lower than the Verwey transition temperature observed for bulk magnetite with the ideal stoichiometry, 122–125 K, which may be explained by several factors, among which are the partial oxidation and small size of the magnetite particles [47,48]. By comparing the magnitude of the magnetic moments of all the A samples, one can observe that A3.2 is magnetically stronger. These results corroborate the XRD and Mössbauer effect data. While goethite, present in A1.1 and A2.1, is antiferromagnetic, having no net magnetization, both magnetite and maghemite are ferrimagnetic presenting sizeable magnetic moments. Moreover, magnetite has a higher magnetic moment than maghemite.

In order to better estimate the values of the blocking temperature and also the kink at lower temperatures, in the samples where the M(T) curves suggest their presence, temperature dependence of AC susceptibility has also been performed for two different frequencies. The data for both the in-phase, χ′(T), and out-of-phase, χ″(*T*) components exhibit the expected superparamagnetic behaviour of nanoparticles systems; χ′(*T*) being easily comparable with the ZFC magnetization curve, while the onset of χ″(*T*) allows to assign with higher precision the blocking temperature of these samples by the occurrence of a maximum at 126, 78.5 and 150 K, for A1.1, A2.1 and A3.2, respectively (right-hand y axis on Figure 6). Additionally, in sample A3.2, a resolved peak at 37.7 K indicates the Verwey transition temperature, which agrees well with the kink observed in the ZFC curve and the presence of magnetite detected by Mössbauer spectroscopy.

**Figure 7:**
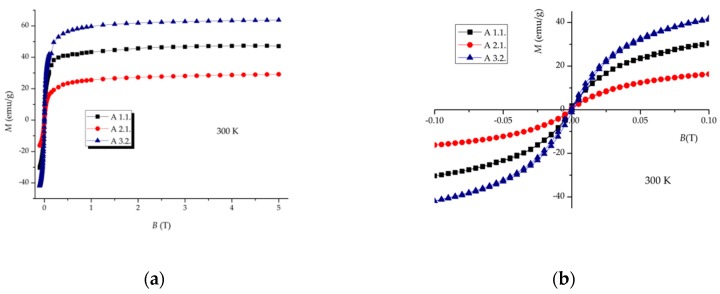

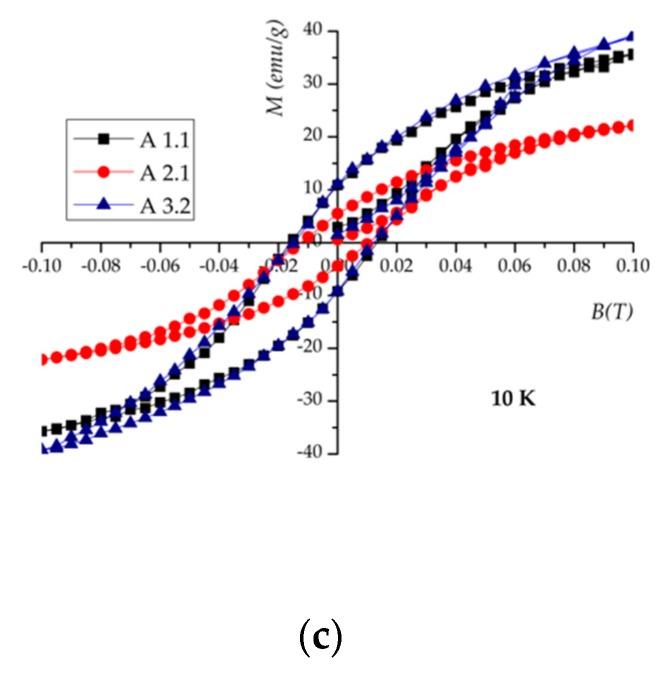
Field dependence of magnetization for the naked SPIONs (Samples A): (**a**) magnetization curve up to 5 T at 300 K, (**b**) hysteresis cycle at 300 K, (**c**) hysteresis cycle at 10 K.

Isothermal magnetization for these naked SPIONs was measured up to 5 T, at fixed temperatures, 10 and 300 K (Figure 7), in order to check the presence of hysteresis and the magnetization saturation for each sample (Table 5). As expected, sample A3.2 shows the highest saturation magnetization reaching the value 64 emu/g. The absence of both coercivity and remanence on the isothermal curves at 300 K of all the A samples (Figure 7) confirms their superparamagnetic behaviour at room temperature while at low temperature, 10 K, a small coercive field, *Hc*, is observed (Figure 7c).

The magnetization curves obtained for coated SPIONs through method 1 (samples B) show *T_B_* values at temperatures lower than those observed for the A samples, as expected considering the smaller size and lower crystallinity of these nanoparticles according to TEM and XRD. Additionally, lower saturation magnetization values were found for both B1.1 and B2.2 by the isothermal curves at 10 and 300 K (Figure S3), confirming the above XRD and Mössbauer data where no spinel type oxide was observed.

**Figure 8:**
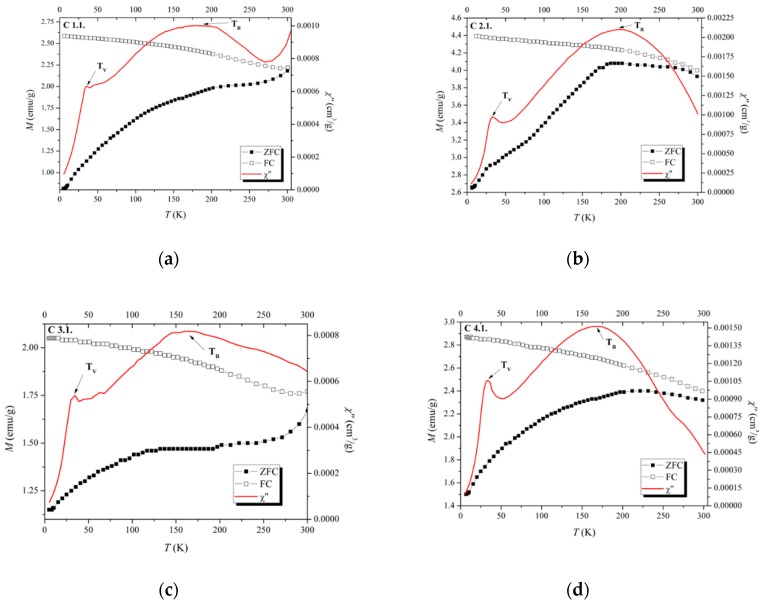
Temperature dependence of magnetization (ZFC/FC curves at 25 G) and AC susceptibility (out-of-phase component χ″ at 995 Hz) of coated SPIONs: (**a**) C 1.1, (**b**) C 2.1, (**c**) C 3.1, (**d**) 4.1.

The magnetic behaviour of the SPIONs coated with different polymers through method 2 (C samples) was put into evidence using both magnetization and AC susceptibility measurements. From ZFC and χ″(T) curves (Figure 8), the smallest *T_B_* = 160 K was found for sample C4.1 coated with the long polymer chain, dextran T70, while the highest, *T_B_* = 195 K, was obtained for C2.1 coated with the smaller polymer chain, dextran T10. It is also important to notice that the Verwey transition temperature, characteristic of magnetite observed in the Mössbauer spectra, was also detected for all C samples, as indicated in Figure 8, by the kink observed in the ZFC magnetization and more accurately determined by the onset of *χ*″(*T*). The narrow range of temperatures where this transition occurs, 33–38 K, is possibly related to the similarly low sizes of the nanoparticles (7.5 and 8.5 nm).

**Figure 9:**
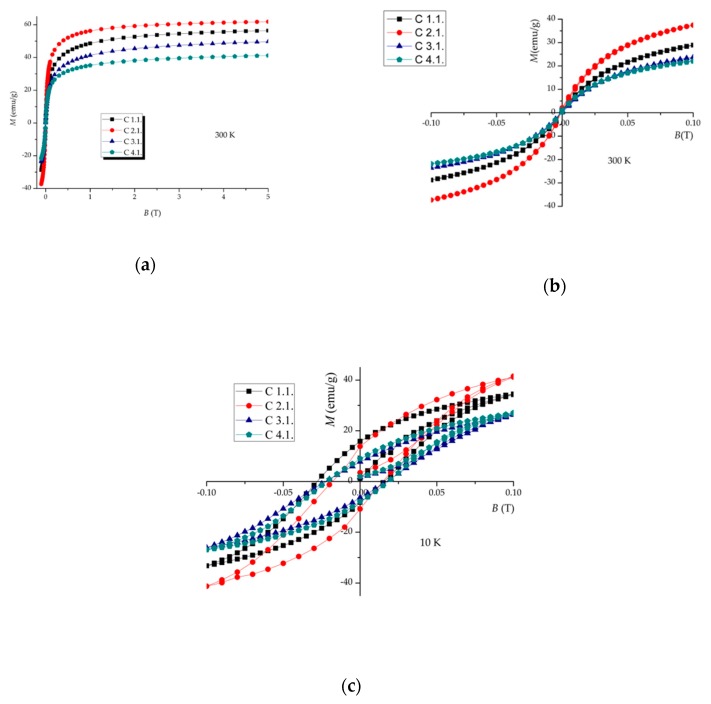
Field dependence of magnetization at 300 K for the naked SPIONs (samples C): (**a**) magnetization curve up to 5 T at 300 K, (**b**) hysteresis cycle at 300 K, (**c**) hysteresis cycle at 10 K.

The same trend found for *T_B_* was also observed for the magnetization saturation values, *Ms*, obtained at 300 K for this set of samples (Figure 9, Table 5). These isothermal magnetization curves show that higher saturation magnetization values are obtained for coated SPIONs with smaller polymer chains, confirming the above M(T) and Mössbauer data. At 10 K, the appearance of a small coercivity is observed in agreement with the characteristics of these nanoparticles being superparamagnetic at room temperature and becoming “blocked” at low temperature, which results in the opening of the hysteresis loop (Figure 9c)). These results indicate that SPIONs coated with smaller polymer chains evidence a stronger magnetism.

Magnetization data show that the blocking temperatures of the nanoparticles in all the samples are below room temperature. The fact that the Mössbauer spectra of some samples is taken at 295 K shows that sextets with very broad peaks are related to the observation time window specific for this technique, determined by the Larmor precession frequency of the nuclear moments around the magnetic moment of the particles. As referred to above, these sextets at 295 K are also typical of the superparamagnetic behavior of oxides that have high magnetic ordering temperatures, above 850 K in the case of bulk maghemite or magnetite. Interactions between the particles magnetic dipoles may also decrease the frequency of the fluctuations of the magnetic dipole directions increasing the relative areas of the sextets with broad peaks.

## 4. Discussion

In the SPIONs’ synthesis protocol, the separation (centrifugation/sedimentation) and drying steps determine the phase composition and consequently the magnetic properties of the precipitated iron oxide phases. Iron oxide NPs separated from the mother solution by sedimentation and/or dried in air, even when centrifuged, evidence poor magnetic properties while those centrifuged and dried in vacuum exhibit a good saturation magnetization value, comparable with the values reported in the literature for magnetite NPs with the same specific size and morphology [47]. These results are explained by the precipitated phases, composition and crystallinity. Iron oxide NPs dried in air are constituted by oxyhydroxides phases, namely antiferromagnetic goethite, and the only ferrimagnetic spinel phase present is maghemite, where all the iron cations are in the 3+ oxidation state. On the other hand, iron oxide NPs centrifuged and dried in vacuum form spinel phases, namely magnetite and maghemite, and no oxyhydroxides are observed. In magnetite domains, part of the iron cations are in the 2+ oxidation state, and the saturation magnetization is higher than that of maghemite.

During SPIONs coating, polymers cannot be added to the synthesis protocol before iron oxides’ precipitation. The addition of polymers during the iron oxide NPs formation inhibits the crystal growth, leading to NPs equal or smaller than 4.5 nm. In this case, Mössbauer and XRD data are consistent with the predominant presence of ferrihydrite and sometimes goethite. These iron oxyhydroxides’ phases have no sizable magnetization and therefore no use for *theranostics*.

However, good results are achieved when the polymers are introduced into the synthesis protocol after the iron oxide precipitation. In this case, the joint analysis of the diffractograms, the Mössbauer spectra (Figure 5) and AC susceptibility (Figure 8), show that when drying is performed in vacuum, the same phases, maghemite and magnetite, are present in both naked and coated SPIONs. Consequently, coated SPIONs have magnetic properties similar to those of the naked SPIONs. Moreover, SPIONs with small polymer chains present higher saturation magnetization values and higher blocking temperatures, closer to those of naked SPIONs.

Although reproducible maghemite-magnetite ratios have been obtained based on synthesis in rare gas [47,49], the SPIONs synthesis protocols followed by our team in previous studies were not reproducible concerning this ratio [35]. In the present study, dehydrating under vacuum and using a final freeze drying step, a procedure that does not require the use of rare gas, the maghemite/magnetite ratios achieved were optimized as well as the magnetization properties of the synthesized samples.

## Figures and Tables

**Table 1:** SPIONs Samples Acronyms.

Sample	Washing	Drying
**A 1.1**	Centrifuged	Air
**A 2.1**	Sedimented	Air
**A 3.2**	Centrifuged	Vacuum

**Table 2:** Coated SPIONs Samples Acronyms.

Sample	Sample Type	Synthesis
**B 1.1**	SPIONs + PEG1000	Method 1
**B 2.2**	SPIONs + Dextran T10	Method 1
**C 1.1**	SPIONs + PEG1000	Method 2
**C 2.1**	SPIONs + Dextran T10	Method 2
**C 3.1**	SPIONs + PEG6000	Method 2
**C 4.1**	SPIONs + Dextran T70	Method 2

**Table 3:** Average (static) diameter and phase composition of naked and coated SPIONs.

Sample	TEMD (nm)	Phases Identified by XRD	Phases Identified by Mossbauer
**A 1.1**	10.5 ± 3.1	goethite spinel	goethite maghemite
**A 2.1**	8.1 ± 1.9	goethite spinel	goethite maghemite
**A 3.2**	7.5 ± 1.5	goethite spinel	goethite maghemite magnetite
**B 1.1**	4.4 ± 1.1	Goethite Ferrihydrite	Goethite Ferrihydrite
**B 2.2**	1.1 ± 0.4	Ferrihydrite	Ferrihydrite
**C 1.1**	7.9 ± 2.3	Spinel	Maghemite Magnetite
**C 2.1**	8.5 ± 1.9	Spinel	Maghemite Magnetite
**C 3.1**	7.6 ± 2.1	Spinel	Maghemite Magnetite
**C 4.1**	7.7 ± 2.0	Spinel	Maghemite Magnetite

**Table 4:** Estimated parameters from the Mössbauer spectra of the naked and coated SPION samples, taken at different temperatures T.

Sample	T	IS	QS	B_hf_ or <B_hf_>	I	Fe Species
A1.1	295 K	0.35	−0.03	25.4	100%	*
A1.1	4 K	0.50 0.45 0.45	0.52 −0.07 −0.25	52.5 53.3 51.4	16% 34% 50%	Fe^3+^ CN = 6 γ Fe_2_O_3_ Fe^3+^ CN = 4 γ Fe_2_O_3_ Fe^3+^ CN = 6 α FeOOH
A2.1	295 K	0.35 0.32	0.60 −0.11	−23.5	32% 68%	*
A2.1	4 K	0.49 0.44 0.47	0.40 −0.10 −0.25	52.7 53.3 51.2	18% 15% 67%	Fe^3+^ CN = 6 γ Fe_2_O_3_ Fe^3+^ CN = 4 γ Fe_2_O_3_ Fe^3+^ CN = 6 α FeOOH
A3.2	295 K	0.33 0.65	0.03 −0.18	30.6 33.3	84% 16%	Fe^3+^ CN = 4, 6 γ Fe_2_O_3_, Fe_3_O_4_ Fe^2.5+^ CN = 6 Fe_3_O_4_
A3.2	4 K	0.49 0.43 0.93	0.02 −0.03 0.12	53.7 51.5 46.2	39% 51% 10%	Fe^3+^ CN = 6 γ Fe_2_O_3_ Fe^3+^ CN = 4, 6 γ Fe_2_O_3_, Fe_3_O_4_ Fe^2+^ Fe_3_O_4_
B1.1	295 K	0.37	0.76	−	100%	Fe^3+^ CN = 6 Ferrihydrite, αFeOOH
B1.1	4 K	0.50 0.49 0.48	0.35 −0.10 −0.25	50.3 46.3 50.5	9% 69% 22%	Fe^3+^ CN = 6 Ferrihydrite Fe^3+^ CN = 6 Ferrihydrite Fe^3+^ CN = 6 α FeOOH
B2.2	295 K	0.37	0.75	-	100%	Fe^3+^ CN = 6 Ferrihydrite
B2.2	4 K	0.50 0.49	−0.04 −0.11	49.5 45.8	36% 64%	Fe^3+^ CN = 6 Ferrihydrite Fe^3+^ CN = 6 Ferrihydrite
C1.1	295 K	0.35 0.64 0.35	0.01 −0.12 0.60	34.7 33.8 −	91% 7% 2%	Fe^3+^ CN=4, 6 γ Fe_2_O_3_, Fe_3_O_4_ Fe^2.5+^ CN = 6 Fe_3_O_4_ Fe^3+^ CN = 4,6 γ Fe_2_O_3_
C2.1	295 K	0.33 0.65 0.36	−0.02 0.02 0.47	34.3 49.1 −	84% 14% 2%	Fe^3+^ CN=4, 6 γ Fe_2_O_3_, Fe_3_O_4_ Fe^2.5+^ CN = 6 Fe_3_O_4_ Fe^3+^ CN = 4,6 γ Fe_2_O_3_
C3.1	295 K	0.37 0.65 0.30	−0.03 0.29 0.66	33.4 33.4 −	90% 7% 3%	Fe^3+^ CN=4, 6 γ Fe_2_O_3_, Fe_3_O_4_ Fe^2.5+^ CN = 6 Fe_3_O_4_ Fe^3+^ CN = 4,6 γ Fe_2_O_3_
C4.1	295 K	0.29 0.64 0.37	0.11 −0.19 0.64	31.8 32.3 −	42% 31% 27%	Fe^3+^ CN=4, 6 γ Fe_2_O_3_, Fe_3_O_4_ Fe^2.5+^ CN = 6 Fe_3_O_4_ Fe^3+^ CN = 4, 6 γ Fe_2_O_3_

IS (mm/s) isomer shift relative to metallic α-Fe at 295 K; QS (mm/s) quadrupole splitting and ε (mm/s) quadrupole shift estimated for quadrupole doublets and magnetic sextets, respectively. B_hf_ (tesla) magnetic hyperfine field; <B_hf_ > average B_hf_ of a distribution; I relative area. Estimated errors ≤0.02 mm/s for IS, QS, ε, <0.2 T for B_hf_ and <4% for I. CN coordination number. * Both the distribution of B_hf_ and the doublet are attributed to Fe^3+^ in γ Fe_2_O_3_ and in α FeOOH, the phases identified at 4 K and by XRD. The doublet is due to the smallest particles with the highest relaxation frequency.

**Table 5:** Magnetic parameters, estimated from the DC magnetization (saturation magnetization, Ms) and AC susceptibility (blocking temperatures, *T_B_*, and Verwey temperatures, *T_V_*) measurements of the naked and coated SPIONs (samples, A, B, and C).

Sample	*M*_s_ * (emu/g) (300 K)	*M_s_* * (emu/g) (10 K)	*T_B_* ** (K)	*T_v_* ** (K)
**A 1.1**	47	56.5	126	-
**A 2.1**	29	36.5	78.5	-
**A 3.2**	64	71	150	37.7
**B 1.1**	~8	13	112	-
**B 2.2**	~1.4	4.5	20	-
**C 1.1**	~56	65.5	~180	35.0
**C 2.1**	~62	69.0	~194	32.8
**C 3.1**	~50	58.0	~165	34.8
**C 4.1**	~41	48.5	~160	32.8

* Magnetization values at 5 T. ** Taken from the maxima observed in χ″(T) at 995 Hz, except for B1.2, and B2.2 where values were roughly determined from ZFC curves.

## Supplementary Materials

The following are available online at https://www.mdpi.com/2079-4991/9/7/943/s1, Figure S1: TEM images of coated SPIONs: (a) B1.1, (b) B2.2., Figure S2: XRD diffractograms and Mössbauer spectra of coated SPIONs (Samples B): (a) B 1.1, (b) B 2.2., Figure S3: Saturation magnetization curves at 300 K (a) and 10 K (b) of coated SPIONs (Samples B).

## References

[1] Kang T., Li F., Baik S., Shao W., Ling D. (2017). Surface design of magnetic nanoparticles for stimuli-responsive cancer imaging and therapy. Biomaterials.

[2] Li R., Liu B., Gao J. (2017). The application of nanoparticles in diagnosis and theranostics of gastric cancer. Cancer Lett..

[3] Lundquist P., Artursson P. (2016). Oral absorption of peptides and nanoparticles across the human intestine: Opportunities, limitations and studies in human tissues. Adv. Drug Deliv. Rev..

[4] Kim J., Mirando A.C., Popel A.S., Green J.J. (2017). Gene Delivery nanoparticles to modulate angiogenesis. Adv. Drug Deliv. Rev..

[5] Lim E.-K., Kim T., Paik S., Haam S., Huh Y.-M., Lee K. (2015). Nanomaterials for Theranostics: Recent Advances and future challenges. Chem. Rev..

[6] Yin H., Kanasty R.L., Eltoukhy A.A., Vegas A.J., Dorkin J.R., Anderson D.G. (2014). Non-viral vectors for gene-based therapy. Nat. Rev..

[7] Thakor A.S., Gambhir S.S. (2013). Nanooncology: The future of cancer diagnosis and therapy. CA Cancer J. Clin..

[8] Bao G., Mitragotri S., Tong S. (2013). Multifunctional nanoparticles for drug delivery and molecular imaging. Annu. Rev. Biomed. Eng..

[9] Mura S., Couvreur P. (2012). Nanotheranostics for personalized medicine. Adv. Drug Deliv. Rev..

[10] Prijic S., Sersa G. (2011). Magnetic nanoparticles as targeted delivery systems in oncology. Radiol. Oncol..

[11] Veiseh O., Gunn J., Zhang M. (2010). Design and fabrication of magnetic nanoparticles for targeted drug delivery and imaging. Adv. Drug Deliv. Rev..

[12] Majidi S., Sehrig F.Z., Samiei M., Milani M., Abbasi E., Dadashzadeh K., Akbarzadeh A. (2016). Magnetic nanoparticles: Applications in gene delivery and gene therapy. Artif. Cells Nanomed. Biotechnol..

[13] Carvalho A., Domingues I., Gonçalves M.C. (2015). Core-shell superparamagnetic nanoparticles with interesting properties as contrast agents for MRI. Mater. Chem. Phys..

[14] Shen Z., Wu A., Chen X. (2017). Iron oxide nanoparticles based contrast agents for magnetic resonance imaging. Mol. Pharm..

[15] Revia R.A., Zhang M. (2016). Magnetite nanoparticles for cancer diagnosis, treatment, and treatment monitoring: Recent advances. Mater. Today.

[16] Usov N.A., Nesmeyanov M.S., Tarasov V.P. (2018). Magnetic vortices as efficient nano heaters in magnetic nanoparticle hyperthermia. Sci. Rep..

[17] Castellani S., Orlando C., Carbone A., Gioia S.D., Conese M. (2016). Magnetofection enhances lentiviral-mediated transduction of airway epithelial cells through extracellular and cellular barriers. Genes.

[18] Zhuang J., Fan K., Gao L., Lu D., Feng J., Yang D., Gu N., Zhang Y., Liang M., Yan X. (2012). Ex vivo detection of iron oxide magnetic nanoparticles in mice using their intrinsic peroxidase-mimicking activity. Mol. Pharm..

[19] Inbaraj B.S., Chen B.-H. (2012). In vitro removal of toxic heavy metals by poly (*γ*-glutamic acid)-coated superparamagnetic nanoparticles. Int. J. Nanomed..

[20] Gao Y., Lim J., Teoh S.-H., Xu C. (2015). Emerging translational research on magnetic nanoparticles for regenerative medicine. Chem. Soc. Rev..

[21] Sneider A., VanDyke D., Paliwal S., Rai P. (2017). Remotely triggered nano-theranostics for cancer applications. Nanotheranostics.

[22] Wadajkar A.S., Menon J.U., Kadapure T., Tran R.T., Yang J., Nguyen K.T. (2013). Design and application of magnetic-based theranostic nanoparticle systems. Recent Pat. Biomed. Eng..

[23] Frank M., Fries L. (1991). The role of complement in inflammation and phagocytosis. Immunol. Today.

[24] Gonçalves M.C. (2018). Sol-Gel Silica Nanoparticles in Medicine: A natural choice. Design, synthesis and products. Molecules.

[25] Gref R., Minamitake Y., Peracchia M.T., Trubetskoy V., Torchilin V., Langer R. (1994). Biodegradable long circulating polymeric nanospheres. Science.

[26] Mitchell R.N., Ratner B.D., Hoffman A.S., Schoen F.J., Lemons J.E. (2004). Innate and adaptive immunity: The immune response to foreign materials. Biomaterials Science: An. Introduction to Materials in Medicine.

[27] Berry C.C., Wells S., Charles S., Curtis A.S.G. (2003). Dextran and Albumin derivatised iron oxide nanoparticles: Influence on fibroblasts in vitro. Biomaterials.

[28] Chouly C., Pouliquen D., Lucet I., Jeune J.J., Jallet P. (1996). Development of superparamagnetic nanoparticles for MRI: Effect of particle size, charge and surface nature on biodistribution. J. Microencapsul..

[29] Gaur U., Sahoo S.K., De T.K., Ghosh P.C., Maitra A., Ghosh P.K. (2002). Biodistribution of fluoreceinated dextran using novel nanoparticles evading reticuloendothelial system. Int. J. Pharm..

[30] Khiabani S.S., Farshbaf M., Akbarzadeh A., Davaran S. (2017). Magnetic nanoparticles: Preparation methods, applications in cancer diagnosis and cancer therapy. Artif. Cells Nanomed. Biotechnol..

[31] García-Jimeno S., Estelrich J. (2013). Ferrofluid based on polyethylene glycol-coated iron oxide nanoparticles: Characterization and properties. Colloids Surf. A.

[32] Illés E., Szekeres M., Tóth I.Y., Farkas K., Földesi I., Szabó A., Iván B., Tombácz E. (2018). PEGylation of superparamagnetic iron oxide nanoparticles with self-organizing polyacrylate-PEG brushes for contrast enhancement in MRI diagnosis. Nanomaterials.

[33] Moghimi S.M., Hunter A.C., Murray J.C. (2001). Long-circulating and target-specific nanoparticles: Theory and practice. Pharmacol. Rev..

[34] Duget E., Vasseur S., Mornet S., Devoisselle J.M. (2006). Magnetic nanoparticles and their applications in medicine. Nanomedicine.

[35] Gonçalves M.C., Fortes L.M., Pimenta A.R., Pereira J.C.G., Almeida R.M., Carvalho M.D., Ferreira L.P., Cruz M.M., Godinho M. (2013). Silica/ormosil SPIONs for biomedical applications. Curr. Nanosci..

[36] Roisnel T., Rodriguez-Carvajal J. (2000). WinPLOTR: A Windows tool for powder diffraction patterns analysis. Mater. Sci. Forum.

[37] Waerenborgh J.C., Rojas D.P., Shaula A.L., Mather G.C., Patrakeev M.V., Kharton V.V., Frade J.R. (2005). Phase formation and iron oxidation state in SrFe(Al)O_3-δ_ perovskites. Mater. Lett..

[38] Hesse J., Rübartsch A. (1974). Model independent evaluation of overlapped Mössbauer spectra. J. Phys. E.

[39] Asadi H., Khoee S., Deckers R. (2016). Polymer-grafted superparamagnetic iron oxide nanoparticles as a potential stable system for magnetic resonance imaging and doxorubicin delivery. RSC Adv..

[40] Khairuddin, Pramono E., Utomo S.B., Wulandari V., Zahrotul W., Clegg F. (2016). FTIR studies on the effect of concentration of polyethylene glycol on polimerization of Shellac. J. Phys. Conf. Ser..

[41] Unterwegen H., Subatzus D., Tietze R., Janko C., Poettler M., Stiegelschmitt A., Schuster M., Maake C., Boccaccini A.R., Alexiou C. (2015). Hypericin-bearing magnetic iron oxide nanoparticles for selective drug delivery on photodynamic therapy. Int. J. Nanomed..

[42] Mørup S., Long G.J. (1987). Mössbauer effect studies of microcrystalline materials. Mössbauer Spectroscopy Applied to Inorganic Chemistry.

[43] Murad E. (1998). Clays and clay minerals: What can Mössbauer spectroscopy do to help understand them?. Hyperfine Interact..

[44] Roca G., Marco J.F., del Puerto Morales M., Serna C.J. (2007). Effect of nature and particle size on properties of uniform magnetite and maghemite nanoparticles. J. Phys. Chem. C.

[45] Predoi D., Kuncser V., Tronc E., Nogues M., Russo U., Principi G., Filoti G. (2003). Magnetic relaxation phenomena and inter-particle interactions in nanosized *γ*-Fe_2_O_3_ systems. J. Phys. Condens. Matter..

[46] Murad E., Johnston J.H., Long G.J. (1987). Iron oxides and oxyhydroxides. Mossbauer Spectroscopy Applied to Inorganic Chemistry.

[47] Goya G.F., Berquó T.S., Fonseca F.C., Morales M.P. (2003). Static and dynamic magnetic properties of spherical magnetite nanoparticles. J. Appl. Phys..

[48] Vandenberghe R.E., Barrero C.A., da Costa G.M., Van San E., De Grave E. (2000). Mössbauer characterization of iron oxides and (oxy)hydroxides: The present state of the art. Hyperfine Interact..

[49] Hong J., Xu D., Yu J., Gong P., Ma H., Yao S. (2007). Facile synthesis of polymer-enveloped ultrasmall superparamagnetic iron oxide for magnetic resonance imaging. Nanotechnology.

