# Supplementary material for: SPIONs Prepared in Air through Improved Synthesis Methodology: the Influence of γ-Fe2O3/Fe3O4 Ratio and Coating Composition on Magnetic Properties
Source: Nanomaterials (Basel). 2019 Jun 28;9(7):943. doi: 10.3390/nano9070943 (PMC6669523; doi:10.3390/nano9070943)
Supplement: Supplementary file 1 [file nanomaterials-09-00943-s001.pdf]

## Supplementary material to:

# SPIONs Prepared in Air through Improved Synthesis Methodology: the Influence of γ-$Fe_2O_3$/$Fe_3O_4$ Ratio and Coating Composition on Magnetic Properties

**Joana C. Matos [1,2,3,*] M. Clara Gonçalves [1,3] Laura C. J. Pereira [2] Bruno J. C. Vieira [2] João Carlos Waerenborgh [2]**

1 Centro de Química Estrutural, Universidade de Lisboa, Av. Rovisco Pais, IST, 1000 Lisboa, Portugal
2 Centro de Ciências e Tecnologias Nucleares, Instituto Superior Técnico, Universidade de Lisboa, 2695-066 Bobadela LRS, Portugal
3 Departamento de Engenharia Química, Instituto Superior Técnico, Universidade de Lisboa, Av. Rovisco Pais, IST, 1000 Lisboa, Portugal
* Correspondence: joana.matos@ist.utl.pt; Tel.: +351-218418137

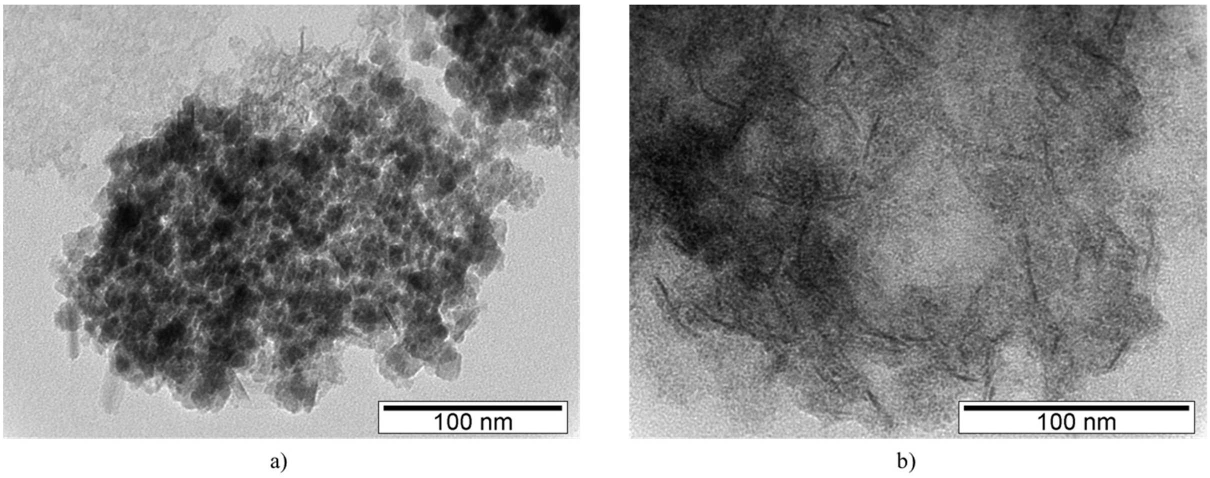


**Figure S1:** TEM images of coated SPIONs: a) B1.1, b) B2.2.

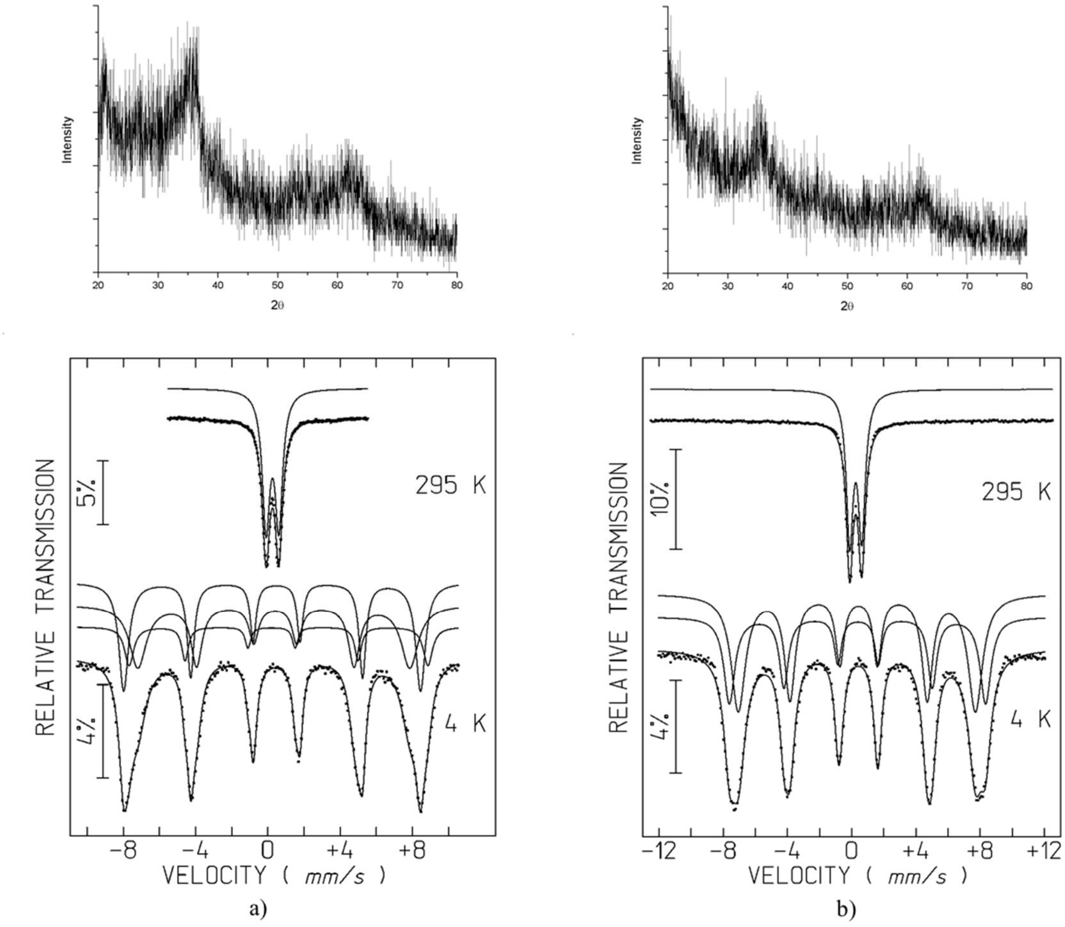


**Figure S2:** XRD diffractograms and Mössbauer spectra of coated SPIONs (Samples B): a) B 1.1, b) B 2.2.

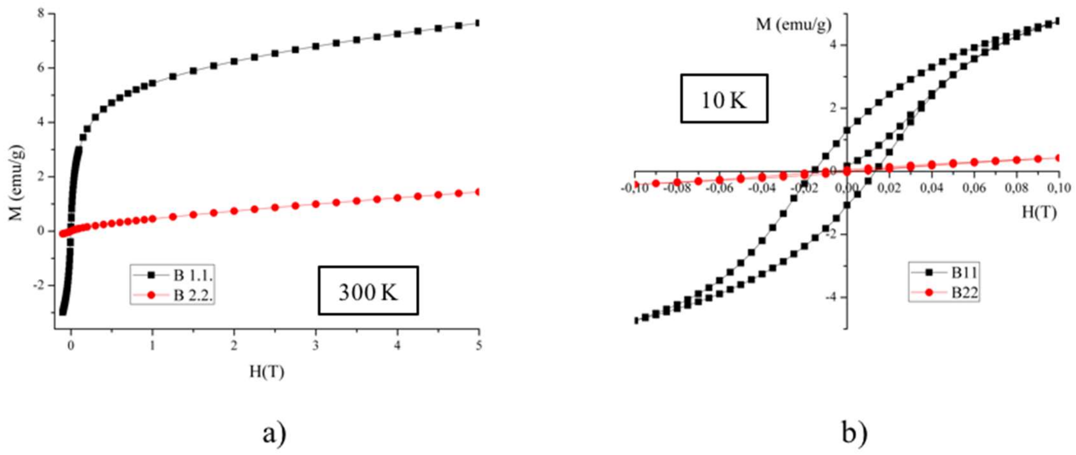


**Figure S3:** Saturation magnetization curves at 300 K (a) and 10 K (b) of coated SPIONs (Samples B)
